# *Lactiplantibacillus plantarum*: a new example of inclusion body producing bacteria

**DOI:** 10.1186/s12934-023-02120-3

**Published:** 2023-06-09

**Authors:** Ricardo Baltà-Foix, Caterina Serrano-Adrover, Adrià López-Cano, Laia Gifre-Renom, Alejandro Sanchez-Chardi, Anna Arís, Elena Garcia-Fruitós

**Affiliations:** 1 Programa de Producció de Remugants, Institut de Recerca i Tecnologia Agroalimentàries (IRTA), Caldes de Montbui, 08140 Spain; 2 Departament de Biologia Evolutiva, Facultat de Biologia, Ecologia i Ciències Ambientals, Universitat de Barcelona, Av. Diagonal 643, Barcelona, 08028 Spain

**Keywords:** *Lactiplantibacillus plantarum*, Inclusion bodies, Recombinant proteins, Active aggregates, Protein aggregation

## Abstract

**Background:**

Lactic Acid Bacteria such as *Lactococcus lactis, Latilactobacillus sakei* (basonym: *Lactobacillus sakei*) and *Lactiplantibacillus plantarum (*basonym: *Lactobacillus plantarum*) have gained importance as recombinant cell factories. Although it was believed that proteins produced in these lipopolysaccharides (LPS)-free microorganisms do not aggregate, it has been shown that *L. lactis* produce inclusion bodies (IBs) during the recombinant production process. These protein aggregates contain biologically active protein, which is slowly released, being a biomaterial with a broad range of applications including the obtainment of soluble protein. However, the aggregation phenomenon has not been characterized so far in *L. plantarum*. Thus, the current study aims to determine the formation of protein aggregates in *L. plantarum* and evaluate their possible applications.

**Results:**

To evaluate the formation of IBs in *L. plantarum*, the catalytic domain of bovine metalloproteinase 9 (MMP-9cat) protein has been used as model protein, being a prone-to-aggregate (PTA) protein. The electron microscopy micrographs showed the presence of electron-dense structures in *L. plantarum* cytoplasm, which were further purified and analyzed. The ultrastructure of the isolated protein aggregates, which were smooth, round and with an average size of 250–300 nm, proved that *L. plantarum* also forms IBs under recombinant production processes of PTA proteins. Besides, the protein embedded in these aggregates was fully active and had the potential to be used as a source of soluble protein or as active nanoparticles. The activity determination of the soluble protein solubilized from these IBs using non-denaturing protocols proved that fully active protein could be obtained from these protein aggregates.

**Conclusions:**

These results proved that *L. plantarum* forms aggregates under recombinant production conditions. These aggregates showed the same properties as IBs formed in other expression systems such as *Escherichia coli* or *L. lactis*. Thus, this places this LPS-free microorganism as an interesting alternative to produce proteins of interest for the biopharmaceutical industry, which are obtained from the IBs in an important number of cases.

## Background

Different recombinant protein production systems were developed with the appearance of recombinant DNA technology [1, 2]. This technology opened a huge spectrum of possibilities, from the obtainment of proteins difficult to isolate from their natural origin to the *de novo* fabrication of polypeptides of interest. Different types of organisms are used for recombinant protein production purposes, including bacteria, yeast, fungi, algae, insect cells and mammalian cells [3]. For proteins that do not need specific post-translational modifications such as glycosylation, bacteria display the best performance-cost ratio as recombinant cell factories. More specifically, *Escherichia coli* has been by far the most used bacterium, due to low production costs, and a myriad of engineering tools that it offers as expression system. Despite all the advantages, the lipopolysaccharides (LPS) present in the outer *E. coli* membrane is an important drawback since the presence of endotoxin or LPS traces in recombinantly produced proteins produces undesirable inflammatory effects. Thus, extra purification steps, which are costly and low-yield procedures, are required to reduce or eliminate LPS from the final recombinant product [4–9]. In this scenario, alternative expression systems such as Lactic Acid Bacteria (LAB), which are LPS-free and are classified as Generally Regarded As Safe (GRAS) organisms by the Food and Drug Administration (FDA), have gained importance. The potential of this group of bacteria has been demonstrated with microorganisms such as *Lactococcus lactis, Latilactobacillus sakei* (basonym: *Lactobacillus sakei*) and *Lactiplantibacillus plantarum (*basonym: *Lactobacillus plantarum*), which have been used as recombinant cell factories [10–19], presenting similar performance to *E. coli* in many cases.

The possibility to produce and isolate recombinant soluble proteins from the cytoplasmic fraction of *L. lactis* is well known, as well as the use of secretion strategies to recover the protein of interest from the culture media [10, 12, 19–22]. Although it is generally accepted that *L. lactis* does not form aggregates during recombinant production processes [12], the existence of protein aggregates or inclusion bodies (IBs) in the cytoplasm of this gram-positive microorganism has been documented [11, 23].

IBs are protein deposits that are generated in recombinant bacteria under overexpression conditions [5]. Their size ranges from 50 to 500 nm, mainly presenting spherical shapes [23–25] and high stability [23, 26–29]. However, some differences in size and surface functional group density have been described in IBs of the same proteins produced by different bacterial systems [26]. Besides, it has been broadly demonstrated that IBs are formed by active forms of the overexpressed protein, which are embedded in an amyloid-like structure [23]. The activity of these protein nanoparticles produced either in *E. coli* or *L. lactis* has led to study their applicability in biotechnology, material sciences and medicine, as biocatalysts [30–32], scaffolds in tissue engineering [24], immunomodulators [28, 29, 33], antimicrobial agents [34], and drug delivery systems in cancer therapy [35–37]. Besides, IBs can be used as a source of soluble and active protein [5]. This strategy becomes very useful for the purification of proteins that are either difficult-to-express (DTE) or prone-to-aggregate (PTA), which present low yields when they are directly isolated from the bacterial cytoplasm in its soluble form.

Although soluble and aggregated forms have been very well-characterized in *E. coli* and in *L. lactis*, they have not been explored so far in other LPS-free systems like *L. plantarum*. Thus, the current work aims to study and characterize the aggregation phenomena in *L. plantarum* to elucidate its capacity to produce IBs and their possible applications. For that, the 39 KDa catalytic domain of bovine metalloproteinase 9 (MMP-9cat) has been used as model protein. It is an enzyme involved in many biological processes such as extracellular matrix degradation, wound healing, angiogenesis, reproduction, growth, and tissue development [28, 38]. Although the whole bovine MMP-9 has not been recombinantly produced so far, its catalytic fragment has been previously produced in *L. lactis* [5, 23] and in *Clearcoli* and it has been described as a PTA protein [13].

## Results

### Solubility of MMP-9cat produced in *L. plantarum*

**Fig. 1:**
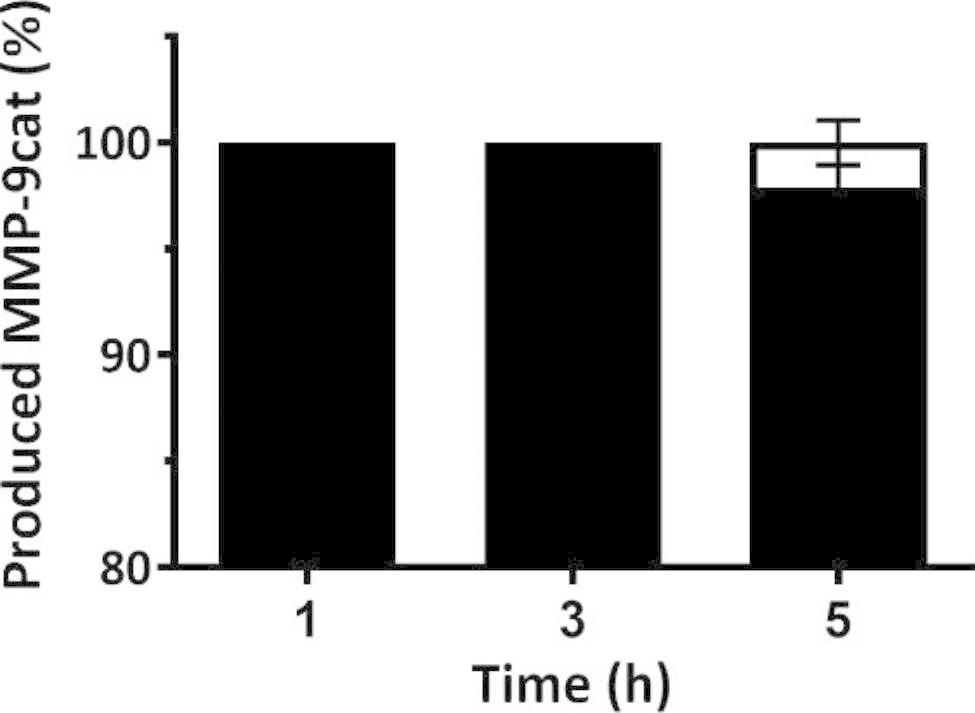
Percentage of aggregation of MMP-9cat produced in *Lactiplantibacillus plantarum* at 1, 3 and 5 h post-induction. The aggregated MMP-9cat is represented in black and the soluble MMP-9cat is represented in white. Error bars indicate the standard error (SE).

To analyze the MMP-9cat production and solubility using *L. plantarum* as cell factory, we determined the percentage of protein in the soluble fraction and in the insoluble (or aggregated) fraction at 1, 3 and 5 h post-induction. The results obtained showed that MMP-9cat produced in *L. plantarum* has a high aggregation rate with 100% of protein aggregated at 1 and 3 h post-induction, and 97.9% ± 1.1 at 5 h post-induction (Fig. 1).

### Characterization of MMP-9cat IBs by electron microscopy

**Fig. 2:**
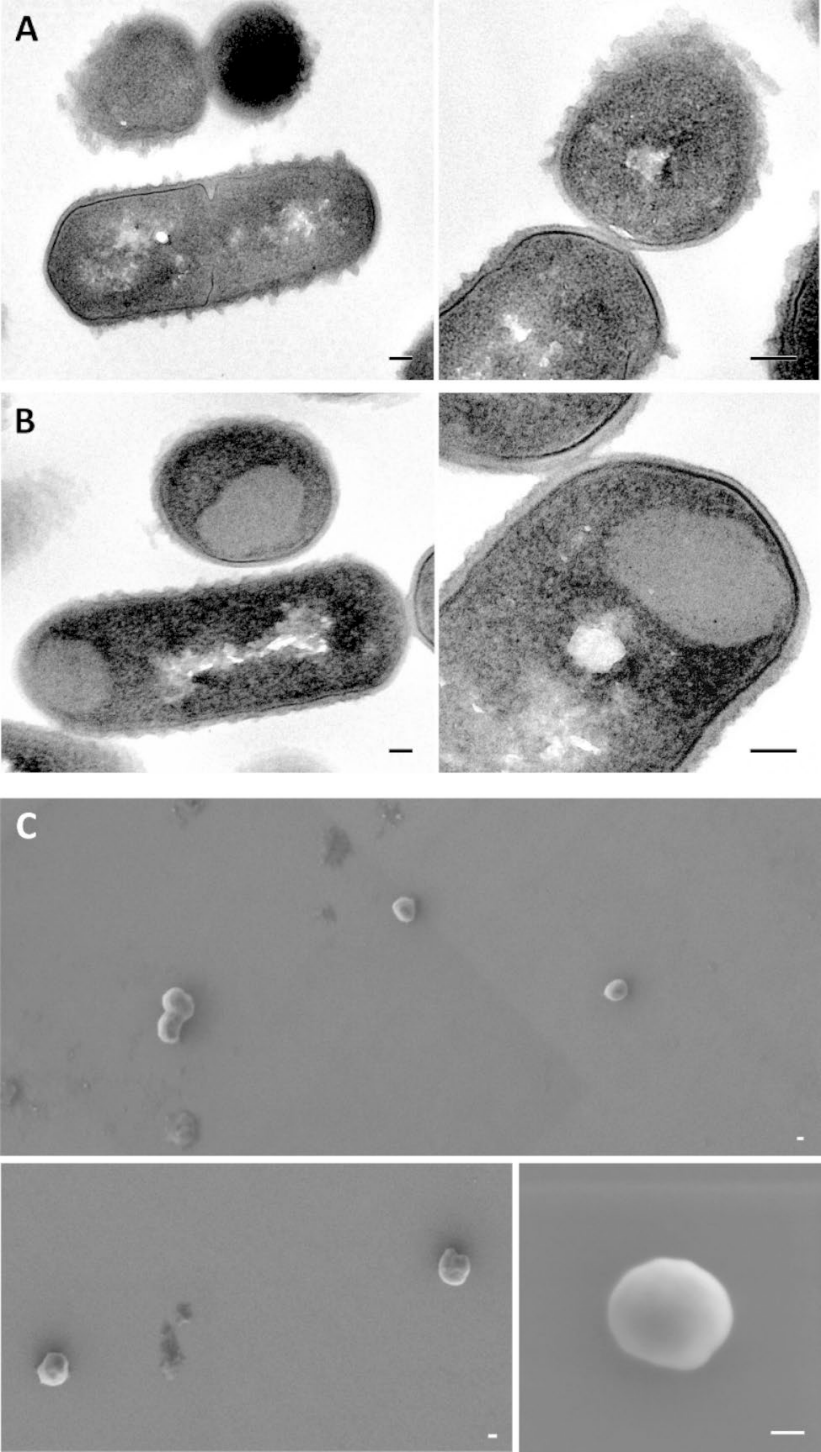
Electron microscopy characterization of MMP-9cat IBs in *Lactiplantibacillus plantarum* and purified MMP-9cat IBs. (**A**) Representative TEM micrographs of ultrastructure of longitudinal and transversal bacilli sections at 0 h post-induction without the presence of MMP-9cat IBs and (**B**) 3 h post-induction with the presence of MMP-9cat IBs. Bars size represents 100 nm. (**C**) Representative FESEM micrographs showing smooth surface, similar size, and round shape of purified MMP-9cat IBs. Bars size represents 100 nm.

When *L. plantarum* cells were analyzed by Transmission Electron Microscopy (TEM), clear electron-dense structures with an average size of 250–300 nm were observed post-induction in the pole of the cells (Fig. 2B). These aggregates observed after induction were not present in TEM micrographs of *L. plantarum* cells at 0 h (Fig. 2A), proving that they were formed during recombinant production.

After production, IBs were purified following standard protocols based on a cell disruption followed by several washing steps with the presence of mild detergents. The ultrastructural morphometry of purified IBs was characterized by Field Emission Scanning Electron Microscopy (FESEM) and micrographs showed smooth and round nanoparticles (Fig. 2C) and confirmed the average size of 250–300 nm shown in TEM micrographs (Fig. 2B).

### Solubilization and purification of MMP-9cat from IBs

**Fig. 3:**
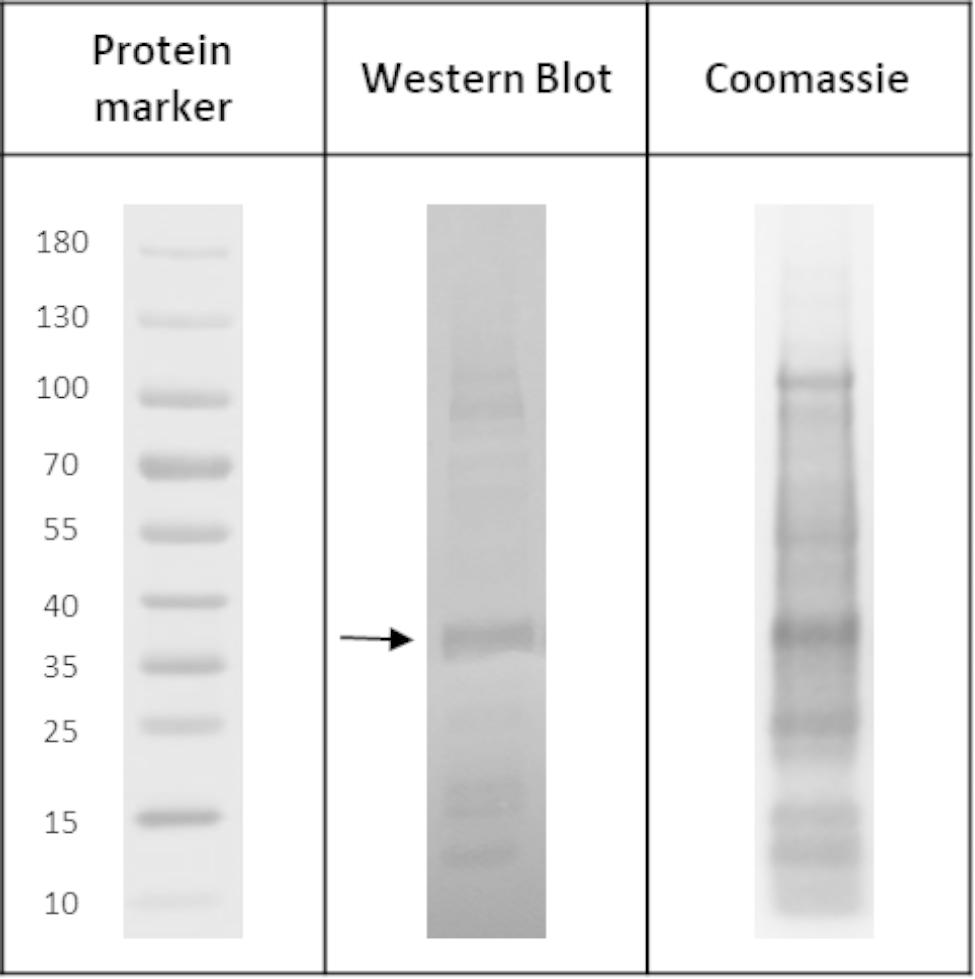
Confirmation of MMP-9cat presence in *Lactiplantibacillus plantarum* IBs by Western Blot with Anti-His antibody and Coomassie staining. The arrow indicates the band with the theoretical size of the protein.

After the characterization of IBs by electron microscopy, the next step was to confirm the presence of MMP-9cat inside the aggregates. Purified IBs were analyzed through Western Blot and Coomassie staining (Fig. 3). The yield and purity were calculated by densitometry analysis (Table 1).

**Table 1:** Yield and purity of purified MMP-9cat IBs produce in *Lactiplantibacillus plantarum.*

	Yield	Purity
MMP-9cat IBs	4.56 mg/L	80.69%

**Fig. 4:**
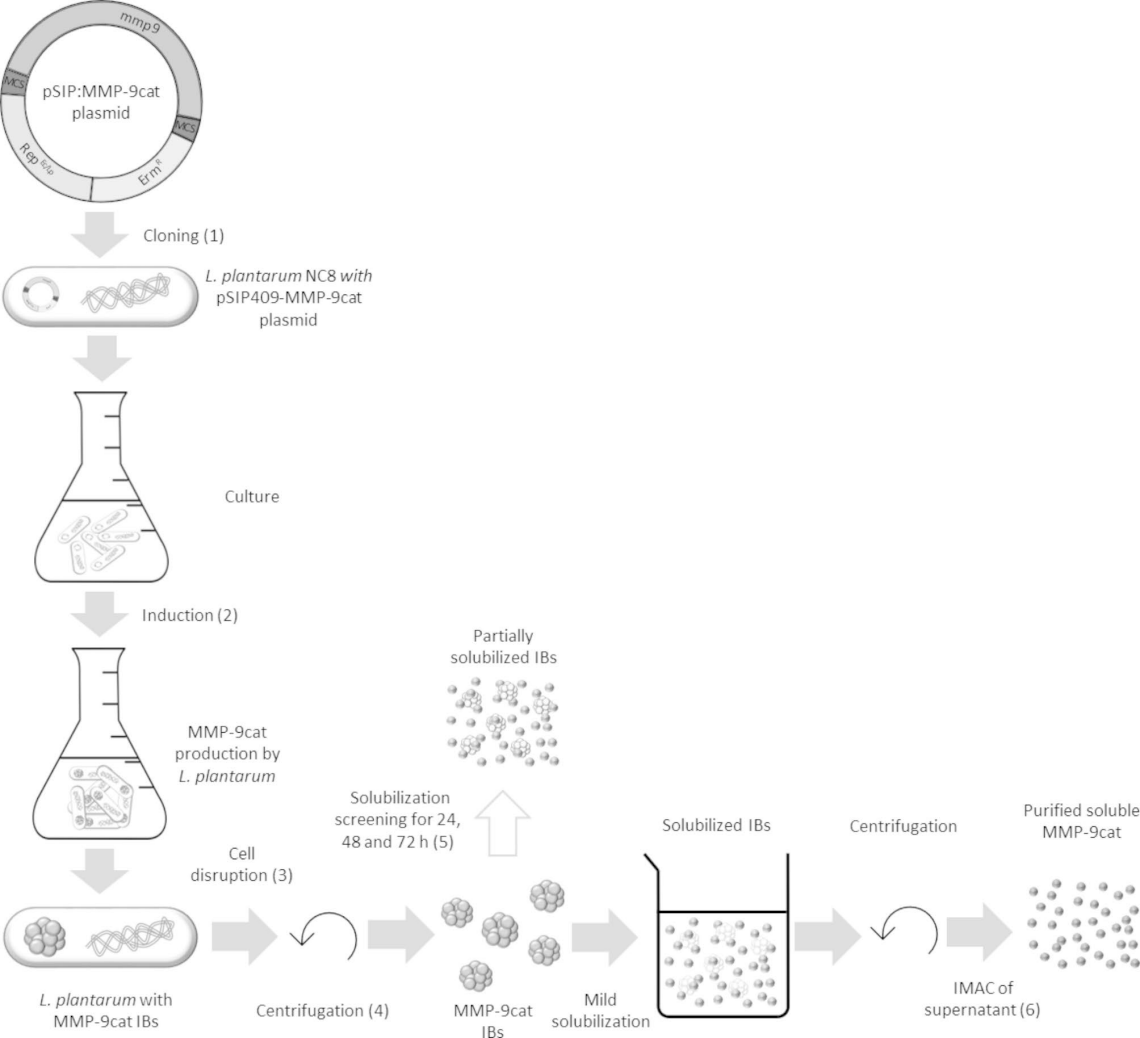
Schematic representation of production, isolation and solubilization of MMP-9cat IBs. *Lactiplantibacillus plantarum* NC8 cells were transformed with the pSIP:MMP-9cat plasmid carrying the catalytic fragment of the bovine MMP-9 (**1**). Cells were grown in MRS medium, and the protein production was induced with sakacin P inducing peptide (SppIP) (**2**). After protein production, *Lactiplantibacillus plantarum* cells were disrupted (**3**) and centrifuged to recover the pellet containing MMP-9cat IBs (**4**). In an intermediate step, IBs were partially solubilized to evaluate their potential activity (**5**). Finally, the pellet was completely solubilized using a non-denaturing process and the soluble protein obtained after a centrifugation process was further purified by immobilized metal affinity chromatography (IMAC) (**6**).

**Fig. 5:**
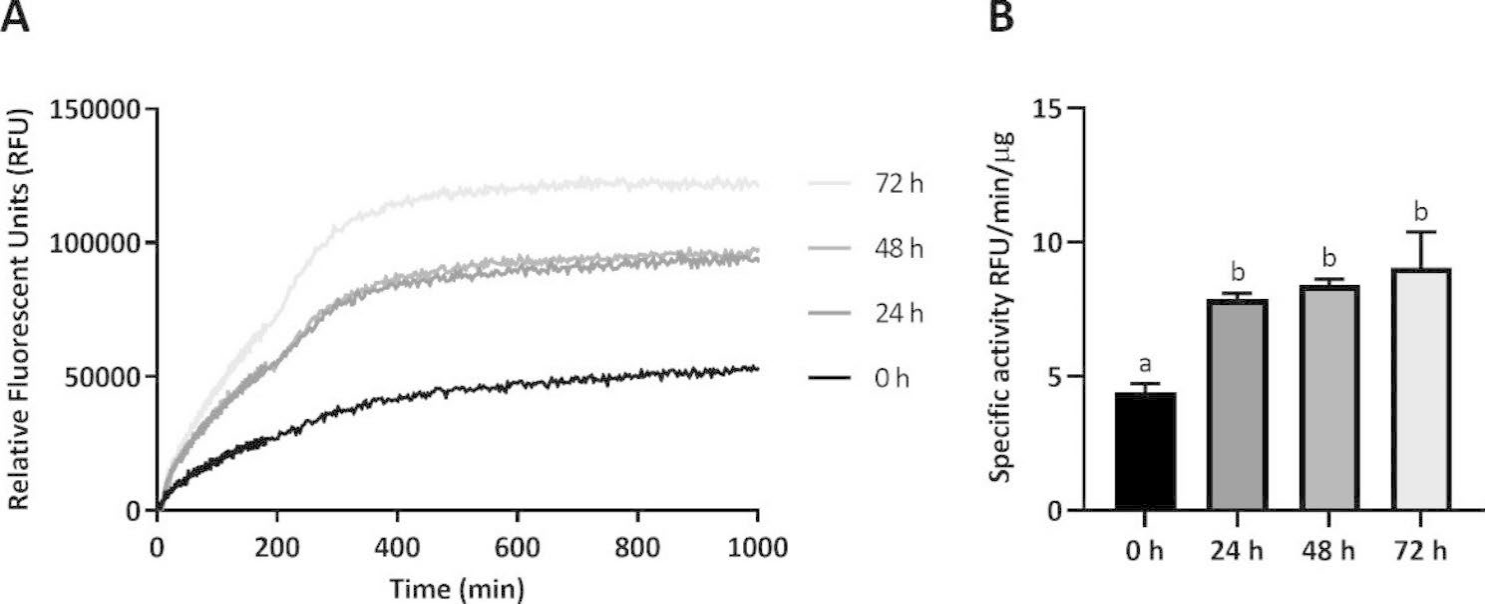
Enzymatic activity of MMP-9cat IBs solubilized. (**A**) MMP-9cat activity kinetics of *Lactiplantibacillus plantarum* MMP-9cat IBs solubilized at RT for 0, 24, 48 and 72 h. (**B**) Specific activity of the solubilized MMP-9cat IBs for 0, 24, 48 and 72 h. Different letters depict significant differences between the samples (*p* < 0.05).

Aiming to determine if the aggregates produced in this recombinant cell factory were constituted of biologically active MMP-9cat, we analyzed their activity after a mild solubilization process. For that, recombinant expression of MMP-9cat in *L. plantarum* cells was induced with 50 ng/mL SppIP and 3 h post-induction, bacterial cells were disrupted by a cell disruptor (Fig. 4). Then, purified IBs were incubated at room temperature (RT) for 0, 24, 48 and 72 h and their activity was determined along the solubilization kinetics of MMP-9cat IBs (Fig. 4 (5), Fig. 5A). The activity assay proved that purified IBs were active, since although they showed activity at 0 h the specific activity significantly increased after the non-denaturing solubilization process (Fig. 5B, p < 0.05).

**Fig. 6:**
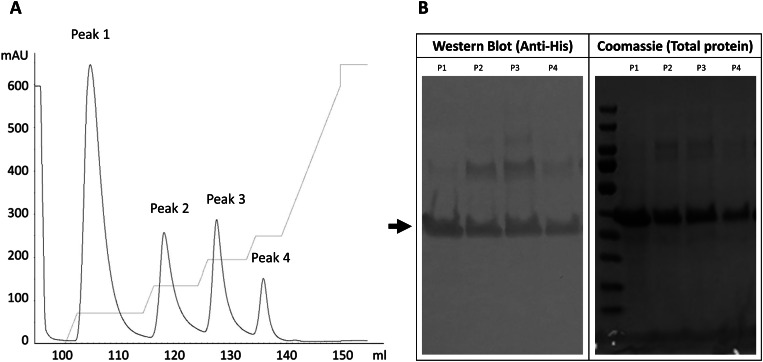
Characterization of soluble MMP-9cat after purification. (**A**) Chromatogram of soluble MMP-9cat with four elution peaks. (**B**) Western Blot with Anti-His antibody and Coomassie staining of the purified protein (P1: peak 1; P2: peak 2; P3: peak 3; P4: peak 4). The black arrow marks MMP-9cat bands.

Taking 48 h as solubilization time, the protein solubilized from IBs was recovered and further purified by affinity chromatography (Fig. 4 (6)) and the soluble protein obtained from the solubilized aggregates was further characterized. The purification chromatogram of the soluble MMP-9cat showed that the protein was eluted in 4 different peaks (Fig. 6A). The final yield and the purity of each peak were quantified by Western Blot and Coomassie staining, respectively (Fig. 6B). All the purified peaks contained pure protein at concentrations ranging from 0.21 to 1.90 mg/L, with peak 1 being the most concentrated (Table 2).

### Soluble MMP-9cat and MMP-9cat IBs specific activity

**Fig. 7:**
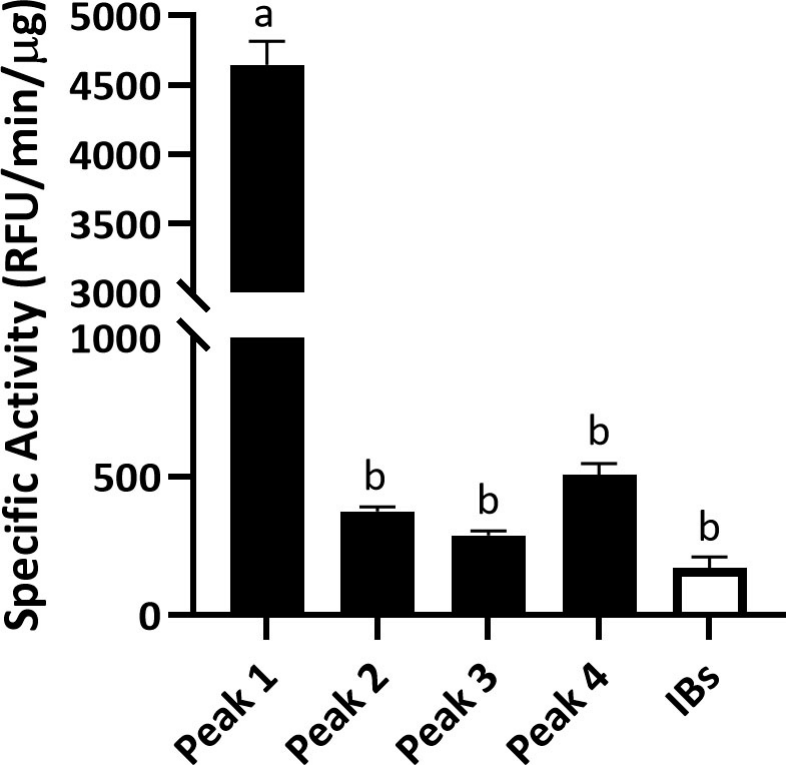
Specific activity of soluble MMP-9cat (1 µg) and MMP-9cat IBs (100 µg). The specific activity of the 4 peaks of soluble MMP-9cat (purified soluble MMP-9cat in Fig. 4) is represented in black and the SA of IBs (MMP-9 cat IBs in Fig. 4) is represented in white. Different letters depict significant differences between samples (*p* < 0.001).

The activity assay proved that soluble MMP-9cat was more active than IBs, which needed much greater concentrations to achieve similar activities (Fig. 7). Peak 1 showed a specific activity higher than peaks 2, 3 and 4 (*p* < 0.001), which did not statistically differ in activity (Fig. 7). The specific activity of the 4 peaks of soluble MMP-9cat was also compared with that of the purified IBs, which presented a specific activity similar to peaks 2, 3 and 4 (Fig. 7). However, it is important to note that amount of IBs used in the assay is higher (100 μg) than that of the soluble version (1 μg).

**Table 2:** Yield and purity of soluble MMP-9cat 4 peaks.

	Yield	Purity
Peak 1	1.92 mg/L	> 99%
Peak 2	0.70 mg/L	> 99%
Peak 3	0.56 mg/L	> 99%
Peak 4	0.21 mg/L	> 99%

## Discussion

Different authors have shown that *L. plantarum* can be used as recombinant expression system, obtaining products for different biological applications [17, 18, 39–43]. The expression of enzymes has been widely explored, obtaining high yields and good enzymatic activities of secreted α-amylases [39], β-galactosidases [18, 41], chitinases [17], reductases [42] or decarboxylases [43]. Moreover, *L. plantarum* has been exploited as life vector for the surface displaying of foreign antigens, being able to induce good immune responses in chickens [44] or mice [45, 46], to recruit T cells [47] or to stimulate monocytes in vitro [48]. Although these studies have proven that *L. plantarum* is an interesting bacterial expression system, the production and aggregation rates of PTA proteins have not been explored so far in this LPS-free bacterial cell factory. *L. plantarum* has been used on some occasions as an alternative to *E. coli*, to avoid or reduce the aggregation of proteins during the recombinant production process [49], but no studies focusing on the possible formation of IBs have been reported so far. Thus, in the present study, we used as model protein the catalytic domain of metalloproteinase 9 (MMP-9cat), which has previously been described to be a protein with a high tendency to aggregate [13, 23]. As previously observed in *L. lactis* [23] and *Clearcoli* [13], when the catalytic domain of MMP-9 is produced in *L. plantarum* practically all the produced protein was found in the aggregated fraction (Fig. 1), which proved that the MMP-9cat’s high tendency to aggregate is not expression system-dependent. When *L. plantarum* cells were analyzed under electron microscopy typical bacilliform cells were observed (Fig. 2A and B) and after the induction of MMP-9cat expression, electron-dense structures were localized in the cell pole (Fig. 2B). Thus, TEM micrographs clearly proved for the first time the formation of intracytoplasmic protein deposits in *L. plantarum* under overproduction conditions, as previously described in *E. coli* [50], *L. lactis* [23], and *P. pastoris* [51]. When these protein aggregates were purified, the morphology of this nanomaterial was analyzed by FESEM (Fig. 2C). The ultrastructure of the protein aggregated formed in *L. plantarum* (Fig. 2C) clearly resembled that of the IBs formed in *E. coli* [25, 52, 53] and *L. lactis* [23, 26], being smooth and round particles within the nanoscale range with a size around 250–300 nm. Although this size is slightly smaller than MMP-9cat IBs formed in *L. lactis*, it is a size in the range of the spectrum of IBs previously described in the literature [54]. Previous studies have shown that production conditions and strain can slightly impact on IB size and shape [23]. Thus, the size and shape of the IBs purified from *L. plantarum* are evidences of the consolidated aggregation process that occurs during the recombinant protein production in this GRAS expression system.

Besides, the protein yield and purity (Fig. 3B) were comparable to other expression systems such as *E. coli* or *L. lactis*. Considering that all the MMP-9cat produced in *L. plantarum* aggregated, the insoluble fraction was further characterized in terms of protein activity. In previous studies, it has been widely demonstrated that IBs are active in their aggregated format [23, 32, 34, 55] and capable to release the protein embedded inside them [56]. Thus, to evaluate the *L. plantarum* IBs characteristics, an initial solubilization kinetics test was done (Fig. 4 (5), incubating IBs for 24, 48 and 72 h. The results showed that this simple solubilization process was enough to observe a significant time-dependent increase in the activity (Fig. 5), which indicated that protein bioavailability increases. That was the first evidence that the proteins embedded in *L. plantarum* IBs were active and the first sign of protein release from these aggregates. This showed that *L. plantarum* IBs have the same properties as protein aggregates formed in other bacterial expression systems, having therefore the potential to be used as a source of soluble protein or as active nanoparticles in their aggregated form for a wide range of applications.

Taking it a step further, the solubilized protein from the IBs using a non-denaturing protocol was purified by affinity chromatography (Fig. 4(6)). Four different MMP-9cat peaks were eluted (Fig. 6A) as described in previous studies where the same protein was produced in *Clearcoli* and *L. lactis* [13]. The yield and purity achieved (> 99%) in the 4 eluted peaks in *L. plantarum* (Fig. 6B and C) was comparable to that observed when MMP-9cat was produced in *L. lactis* [13]. However, in *Clearcoli* the purity and yields of the protein solubilized found in a previous study were lower [13]. In *L. plantarum*, the protein activity differed among the 4 peaks (Fig. 7). Peak 1 of soluble protein showed an activity higher than that observed with peaks 2, 3 or 4, which showed similar levels among them (Fig. 7). The same profile was observed in a previous study in *L. lactis*, where peak 1 showed the highest activity values [13]. In *Clearcoli*, however, peak 2 was the most active one and peaks 1, 3 and 4 showed just residual activity [13]. In terms of activity values, peak 1 of *L. lactis* [13] showed an activity approximately 2-fold higher than the presented by peak 1 in *L. plantarum* in this study (Fig. 7). In the case of *Clearcoli*, the activity values of peak 2, which was the most active one, [13] was significantly lower than those shown by *L. plantarum* and *L. lactis* [13]. Differences of activity observed in the different elution peaks could be attributed to different conformations of the protein eluted in each specific peak, indicating that proteins with different conformations are coexisting in the same protein aggregate.

We could also observe that the activity of MMP-9cat IBs produced in *L. plantarum* was much lower than that of the soluble version (Fig. 7). The amount of IBs analyzed in Fig. 7 were 100 μg while only 1 μg of the soluble protein was used, which meant that an amount 100-time higher of IBs is needed to reach the activity value of 1 μg of soluble MMP-9cat (Fig. 7). This pattern is comparable to that seen in *E. coli* and *L. lactis* with other proteins [13, 57] showing that the protein embedded in IBs has different conformations and activities. Besides, it has already been described that although IBs have lower activity than their soluble counterpart, they have a huge potential when administered in vivo due to their stability and their capacity to slowly release the protein of interest [58].

Altogether we proved that *L. plantarum* IBs possess the same properties as protein aggregates formed in other bacterial expression systems, having the potential to be used as a source of soluble protein or as active nanoparticles in their aggregated form for a wide range of applications in biotechnology and biomedicine. The use of IBs as a source of soluble protein is an extremely useful approach when the protein of interest is not produced in a soluble form. These proteins can be easily purified in an active and highly pure form through a simple solubilization process, which has proven to be effective also for *E. coli* [34] and *L. lactis* [5] IBs. Therefore, *L. plantarum* arises as a new endotoxin-free environment system to isolate IBs to obtain soluble and functional proteins in any interested industry, which so far only uses *E. coli* IBs as the starting material to produce an important percentage of the commercialized recombinant proteins [59].

## Conclusions

These results demonstrated the aggregation process in *L. plantarum* in general. The IBs produced by this expression system are another example that protein aggregation is not a specific phenomenon occurring only in *E. coli* and *L. lactis.* Moreover, the enzymatic activity of *L. plantarum* IBs has been demonstrated and, in consequence, this broadens the catalogue of possibilities to produce proteins of industrial interest using IBs as a source of soluble protein. Thus, *L. plantarum* gains relevance as a promising LPS-free recombinant protein expression alternative to *E. coli*.

## Methods

### Strains and media

The bacterial strains used in this study were *Lactococcus lactis subsp*. *cremoris* NZ9000 [60], *Escherichia coli* JM110 (Stratagene, Ref: 200239-11) and *Lactiplantibacillus plantarum* NC8 (CCUG 61,730, Culture Collection University of Gothenburg, Sweden) [61]. *L. lactis* was grown in M17 media (Merck, Ref: 1,151,080,500) supplemented with 0.5% glucose at 30 °C without shaking. *E. coli* was grown in Luria-Bertrani media (10 g/L tryptone, 5 g/L yeast extract, 10 g/L NaCl) at 37 °C with continuous shaking at 250 rpm. *L. plantarum* was grown at 30 °C without shaking in MRS media without glucose (Conda, Ref: 1295) supplemented with 2% glucose when necessary.

### Plasmids and cloning

The part of the gene codifying for the catalytic domain of bovine metalloproteinase 9 (MMP-9cat) (*mmp9* gene fragment; Phe107-Pro449 NM_174744)) was obtained from *L. lactis*/pNZ8148:MMP-9cat (Cm^R^, chloramphenicol resistant) previously developed by our group [5, 23]. Plasmid pSIP409 (Erm^R^, erythromycin resistant), kindly provided by Dr. Lars Axelsson [14], was used to clone *mmp9* gene fragment by replacing β-glucuronidase gene (*gus*), flanked by *NcoI* and *XbaI* restriction sites. Briefly, pSIP409 plasmid was transformed into *E. coli* JM110. For the isolation of both plasmids pNZ8148:MMP-9cat and pSIP409 the Spin Miniprep Kit (Qiagen, Ref: 27,104) was used. For the purification of pNZ8148:MMP-9cat from *L. lactis* [5] an extra lysis step adding 10 mg/mL of lysozyme in the lysis buffer P2 for 1 h at 37 °C was added being necessary for Gram-positive bacteria. Both plasmids (pNZ8148:MMP-9cat and pSIP409) were digested by FastDigest *NcoI* and *XbaI* restriction enzymes (ThermoScientific, Ref: FD0574 and FD0684) for 1 h at 37 °C. The *mmp9* gene fragment obtained after this digestion was ligated into digested pSIP409 (pSIP:MMP-9cat) by T4 DNA ligase (ThermoFisher, Ref: EL0011) for 1 h at 22 °C. For this, 500 ng of vector and a 3:1 insert:vector ratio were maintained within a final volume of 20 µL. For its transformation in *L. plantarum*, ligation product was purified to eliminate ligation buffers and T4 enzyme using the Spin Miniprep columns (Qiagen), starting the procedure after the lysis step.

For *L. plantarum* electrocompetent cell preparation, cells were grown in MRS broth without glucose with 1% glycine at 30 °C overnight (O/N) in anaerobic conditions. The O/N culture was inoculated in fresh MRS media with 2% glucose and 1% glycine at an optical density (OD_600nm_) of 0.25. The culture was incubated anaerobically at 30^o^C until the OD_600nm_ reached 0.6. The culture was harvested and chilled on ice for 20 min before starting washes. Then, the culture was centrifuged at 4,000 x *g* for 15 min at 4 °C followed by two washes with an equal volume to that harvested, first with ice-cold 1mM MgCl_2_ and second with ice-cold 30% polyethylene glycol 1500 (PEG-1500). Finally, competent cells were resuspended in 1/100 initial volume of PEG-1500.

The transformation of *L. plantarum* was performed following the protocol described by Aukrust and Blom [62]. Briefly, 0.24 µg of the ligation product were added in 40 µL of electrocompetent cells and it was electroporated using a Bio-Rad GenePulser under the following electrical conditions: 1,500 V, 400 Ω, and 25 µF in pre-cooled 2 mm electroporation cuvettes. After the pulse, cells were restored in 1 mL of MRS with 2% glucose for 2 h at 30 °C [62] and plated in MRS 2% glucose agar with 10 µg/mL Erm for selection of transformants.

### MMP-9cat production in *Lactiplantibacillus plantarum*

*L. plantarum* hosting pSIP:MMP-9cat was cultured O/N under the conditions previously described. The O/N culture was inoculated in 200 mL shake flasks at OD_600nm_ = 0.1. MMP-9cat production was induced by adding 50 ng/mL of sakacin P inducing peptide (SppIP) (GenScript, Sequence: MAGNSSNFIHKIKQIFTHR) [14] when the cultures reached OD_600nm_ = 0.3. After the induction, cultures were grown for 5 h and 25 mL samples were taken at 0, 1, 3, and 5 h post-induction. Erm was used for plasmid maintenance at 10 µg/mL.

Samples were harvested by centrifugation at 6,000 *x g*, 4 °C for 15 min. Pellets were suspended in 0.5 mL PBS 1X, jacketed in ice and disrupted by sonication with Branson Digital Sonifier SFX 550 for 3 rounds (0.5 s ON/0.5 s OFF, total time 1.5 min, 10% A). After, soluble and insoluble fractions were separated by centrifugation at 15,000 *x g*, 4 °C for 15 min. Insoluble pellets were suspended in 0.5 mL PBS 1X when needed for protein determination. The experiment was done by triplicate.

### Protein solubility determination

The soluble and insoluble protein fractions were analyzed by Western Blot. A 15% denaturing sodium dodecyl sulfate polyacrylamide gel electrophoresis (SDS-PAGE) was prepared. All samples were resuspended in PBS with Laemmli loading buffer (100 mM Tris base, 8% glycerol, 55 mM SDS, 4% β-mercaptoethanol, 1.6 M urea). Soluble and insoluble (inclusion bodies) fractions were boiled for 10 and 40 min before electrophoresis, respectively. Protein bands were electroblotted into polyvinylidene difluoride (PVDF) membranes at 2.5 A and 25 V for 10 min, followed by a blocking step with BSA O/N at 4 °C (5% BSA in TBST buffer: 10 mM Tris, 150 mM NaCl, 0.05% Tween 20). Anti poly-histidine (Santa Cruz Biotechnology; mouse) was used as the primary antibody at a 1/1,000 dilution in BSA-TBST buffer, in which membranes were incubated for 2 h at RT, followed by 3 washes in TBST buffer. Then, membranes were incubated in a 1/20,000 dilution in TBST of an anti-mouse IgG-alkaline phosphatase (Sigma), used as secondary antibody, along 1 h at RT followed by 3 washes in TBST buffer. Protein bands were developed after adding the alkaline phosphatase substrate solution NBT/BCIP (Thermo Scientific). A protein marker, PageRuler™ Prestained Protein Ladder, has been loaded in all the gels (ref. 26,616, ThermoFisher Scientific). Bands were quantified with a standard curve of purified soluble MMP-9cat previously produced in *L. lactis* [5] Densitometry analyses were performed with ImageJ software.

### MMP-9cat Inclusion Bodies production and purification in *Lactiplantibacillus plantarum*

Cultures of 1 L (1 L of media in 1 L bottles) of *L. plantarum*/pSIP:MMP-9cat were grown at 30 °C without agitation and induced with 50 ng/mL SppIP at an OD_600nm_ = 0.3. The whole volume was recovered after 3 h of production and centrifuged at 6,000 *x g* for 15 min at 4 °C. Pellets of 500 mL of culture were suspended in 300 mL of PBS 1X with EDTA-free protease inhibitor cocktail (Roche). Bacteria were mechanically disrupted by a cell disruptor (Constant Systems Ltd) with 2 cycles at 40 kpsi. Then, a freeze/thaw cycle was performed O/N at -80 °C. After, lysozyme (50 mg/mL) at 0.01 mg/mL and mutanolysin (1,000 U/mL) at 1 U/mL were added and samples were incubated for 2 h at 37 °C and 250 rpm. Just after that, samples were frozen at -80 °C O/N and thawed the next day. Then, samples were washed with 4 µL/mL Triton X-100 for 1 h at RT and 100 µL of sample were plated onto MRS without glucose agar plates supplemented with 2% glucose. Plates were incubated O/N at 30 °C to evaluate the presence of viable cells. If bacterial colonies were observed, extra freeze/thaw cycles were performed until complete elimination of viable bacteria. Once no colonies were observed, the samples were washed in 0.25 µL/mL Nonidet P40 for 1 h at 4 °C. After, they were then treated with 6 µL/mL of 0.1 M MgSO_4_ and 6 µL/mL of 1 mg/mL DNase and incubated at 37 °C and 250 rpm for 1 h. Finally, samples were centrifuged at 6,000 *x g* for 30 min. Each pellet, containing IBs, of 50 mL of culture was resuspended in 5 mL of lysis buffer (Tris 50 mM, NaCl 100 mM, 1 mM EDTA) with added 0.05% Triton X-100. Once suspended, samples were frozen O/N at -80 °C and thawed the next day. After thawing, samples were centrifuged at 6,000 *x g* for 30 min and suspended in 5 mL of PBS. Finally, pure IBs were obtained after a centrifugation step at 15,000 *x g* for 15 min discarding the supernatant. IBs were quantified by Western Blot using the protocol described in “Protein solubility determination”.

### Electron Microscopy

Ultrastructural characterization of intracellular and isolated IBs was performed with two high-resolution electron microscopy techniques. Imaging of intracellular IBs was performed with standard Transmission Electron Microscopy (TEM) procedures adapted to this type of sample [23, 58, 63, 64]. Briefly, pellets of bacilli with and without IBs were fixed with 2.5% glutaraldehyde in 0.1 M phosphate buffer (PB) at pH 7.2, postfixed in 1% osmium tetroxide containing 0.8% potassium ferrocyanide in PB, dehydrated in acetone, embedded in Spurr resin and polymerized at 60 °C during 48 h. Ultrathin Sect. (70 nm) obtained with an ultramicrotome UCT7 (Leica Microsystems) were placed in Cu grids (200 mesh) and contrasted following routine protocol of uranyl acetate and lead citrate solutions. Samples were observed in a TEM JEM 1400 (Jeol) equipped with an Erlangshen CCD camera (Gatan) and operating at 80 kV.

Ultrastructural morphometry (size and shape) of nanoparticles was performed and characterized at nearly native state with field emission scanning electron microscopy (FESEM). Drops of 20 µL of IBs sample were directly deposited on silicon wafers (Ted Pella Inc.) for 30 s and immediately observed without coating with a FESEM Merlin (Zeiss) operating at 1 kV and equipped with a high-resolution secondary electron detector.

### Solubilization of MMP-9cat from IBs

MMP-9cat IBs were resuspended in Tris glycerol buffer (20 mM Tris HCl pH = 8 and 5% glycerol) at a concentration of 2 mg/mL. Aliquots of 50 µL containing 100 µg of MMP-9cat IBs were solubilized at RT for 0 h, 24 h, 48 and 72 h. After solubilization, the mixture was recovered to determine the activity kinetics and the specific activity (SA) of the partially solubilized MMP-9cat IBs, containing a mixture of non-solubilized IBs and solubilized protein.

### Production and purification of the solubilized MMP-9cat

A culture of *L. plantarum*/pSIP:MMP-9cat was induced for 3 h. The whole volume was centrifuged at 6,000 *x g* for 15 min and 4 °C, and the pellets were frozen at -80 °C. Pellets of 500 mL of culture were resuspended in 30 mL of PBS with EDTA-free protease inhibitor cocktail (Roche). Samples were subjected to 4 rounds of French Press disruption at 1,500 psi, intercalated by a minimum of 5 min repose in ice. After that, 0.05 mg/mL lysozyme (50 mg/mL) was added and samples were incubated for 2 h at 250 rpm and 37 °C. The mixture was centrifuged at 15,000 *x g* for 45 min and 4 °C, the supernatant was discarded and the pellet was suspended in the same volume of H_2_O and centrifuged again at 15,000 *x g* for 15 min and 4 °C. Pellets were weighted and solubilized in 0.2% N-lauroyl sarcosine and 40 mM Tris solution at a ratio 1:40 (g:mL) for 48 h at RT under agitation. The supernatant was recovered through centrifugation at 15,000 x *g* for 45 min at 4 °C.

NaCl and imidazole were added to the supernatant to equilibrate the samples with the binding buffer composition (20 mM Tris pH = 8, 500 mM NaCl, 20 mM Imidazole). After that MMP-9cat was purified by immobilized metal affinity chromatography (IMAC) in an ÄKTA purifier FPLC (GE Healthcare) using 1 mL HisTrap HP columns (GE Healthcare). The selected fractions were dialyzed in Tris Glycerol buffer (20 mM Tris HCl pH = 8, 5% glycerol) O/N at 4 °C and with gentle agitation. The amount of purified protein was determined by UV absorbance measured with Nanodrop 1000 Spectrophotometer (Thermo Fisher Scientific). The integrity of the proteins was analyzed by SDS-PAGE.

### MMP-9cat purity determination

The purity of the soluble MMP-9cat purified by IMAC and the purified MMP-9cat IBs was determined by SDS-PAGE following a Coomassie staining (Bio-Rad Laboratories) of the gel. After the electrophoresis, the gel was stained for 1 h with gentle agitation. Then, the excess stain was removed with distaining solution (30% v/v methanol and 7% v/v acetic acid in distilled water). Densitometry analyses by ImageJ software were performed for purity calculation. Coomassie-stained bands were compared with bands obtained by WB using the protocol described in “Protein solubility determination“. Coinciding bands in two techniques were considered as MMP-9cat and the rest of the bands stained were considered impurities. The purity percentage was calculated by dividing the densitometry outcomes of the bands of interest by the densitometry outcomes of all bands.

### Activity of the solubilized MMP-9cat, MMP-9cat IBs and partially solubilized MMP-9cat IBs

The enzymatic activity of MMP-9 catalytic domain was determined by DQ gelatin^™^ assay (Invitrogen). Solubilized MMP-9cat samples and MMP-9cat IBs samples were diluted in Tris Glycerol buffer (20 mM Tris HCl pH = 8, 5% glycerol) at specific concentrations depending on the samples. Soluble MMP-9cat samples were diluted at a concentration of 0.02 mg/mL and MMP-9cat IBs samples were diluted at a concentration of 2 mg/mL. Partially solubilized MMP-9cat IBs were totally recovered after solubilization times of 0, 24, 48 and 72 h. The concentration of MMP-9cat IBs before solubilization was 2 mg/mL. After samples preparation, 100 µL of Assay buffer (50 mM Tris HCl pH = 7.4, 150 mM NaCl, 5 mM CaCl_2_, 0.01% Tween 20) were loaded in an opaque 96-well plate with transparent bottom. Then 50 µL containing either 1 µg of solublilized MMP-9cat or 100 µg of MMP-9cat IBs or partially solubilized MMP-9cat IBs were plated by triplicate. Finally, 50 µL of 0.005 mg/mL dye-quenched gelatin solution were added to each well. The degradation of the gelatin (emission of fluorescence) was measured in a Victor3^™^ Plate Reader (PerkinElmer’s) at 495/515 nm (excitation/emission wavelengths) every 30 s for 2 h for SA calculation, or every 3 min for 16 h for activity kinetics measurement. The SA of soluble MMP-9cat, MMP-9cat IBs and partially solubilized MMPcat IBs was extracted from the kinetic data, by obtaining the initial velocity (relative fluorescence units per minute, rfu/min) for each µg of MMP-9cat in the wells (rfu/min/µg). A model using R was done to adjust data.

### Statistics

SA experiments were analyzed by ANOVA and Tukey’s multiple comparisons test (GraphPad) with different letters depicting significant differences between samples. All experiments were performed in triplicate and represented as the mean of non-transformed data ± non-transformed standard error of the mean (SEM).

## Data Availability

All data analyzed during the current study are available from the corresponding author on reasonable request.

## References

[1] Ferrer-Miralles N, Domingo-Espín J, Corchero J, Vázquez E, Villaverde A (2009). Microbial factories for recombinant pharmaceuticals. Microb Cell Fact.

[2] Saccardo P, Corchero JL, Ferrer-Miralles N (2016). Tools to cope with difficult-to-express proteins. Appl Microbiol Biotechnol Appl Microbiol Biotechnol.

[3] Gifre L, Arís A, Bach À, Garcia-Fruitós E (2017). Trends in recombinant protein use in animal production. Microb Cell Fact.

[4] European Medicines Agency. ICH guideline Q4B Annex 14 to note for evaluation and recommendation of pharmacopoeial texts for use in the ICH regions on bacterial endotoxins tests – general Chap. 2013.

[5] Gifre-Renom L, Cano-Garrido O, Fàbregas F, Roca-Pinilla R, Seras-Franzoso J, Ferrer-Miralles N (2018). A new approach to obtain pure and active proteins from *Lactococcus lactis* protein aggregates. Sci Rep.

[6] Taguchi S, Ooi T, Mizuno K, Matsusaki H (2015). Advances and needs for endotoxin-free production strains. Appl Microbiol Biotechnol.

[7] Wakelin SJ, Sabroe I, Gregory CD, Poxton IR, Forsythe JLR, Garden OJ (2006). Dirty little secrets”-Endotoxin contamination of recombinant proteins. Immunol Lett.

[8] McCullough KZ. The Bacterial Endotoxins Test: A Practical Approach. 2011.

[9] Braude AI (1964). Bacterial endotoxins. Sci Am.

[10] Morello E, Bermúdez-Humarán LG, Llull D, Solé V, Miraglio N, Langella P (2007). *Lactococcus lactis*, an efficient cell factory for recombinant protein production and secretion. J Mol Microbiol Biotechnol.

[11] Cano-Garrido O, Rueda FL, Sànchez-García L, Ruiz-Ávila L, Bosser R, Villaverde A (2014). Expanding the recombinant protein quality in *Lactococcus lactis*. Microb Cell Fact.

[12] Song AAL, In LLA, Lim SHE, Rahim RA (2017). A review on Lactococcus lactis: from food to factory. Microb Cell Fact.

[13] Carratalá JV, Gifre-Renom L, Roca-Pinilla R, Villaverde A, Arís A, Garcia-Fruitós E (2021). Selecting subpopulations of high-quality protein conformers among conformational mixtures of recombinant bovine mmp-9 solubilized from inclusion bodies. Int J Mol Sci.

[14] Sørvig E, Mathiesen G, Naterstad K, Eijsink VGH, Axelsson L (2005). High-level, inducible gene expression in Lactobacillus sakei and *Lactobacillus plantarum* using versatile expression vectors. Microbiology.

[15] Sørvig E, Grönqvist S, Naterstad K, Mathiesen G, Eijsink VGH, Axelsson L (2003). Construction of vectors for inducible gene expression in *Lactobacillus sakei* and *L. plantarum*. FEMS Microbiol Lett.

[16] Maldonado A, Ruiz-Barba JL, Jiménez-Díaz R (2003). Purification and genetic characterization of plantaricin NC8, a novel coculture-inducible two-peptide bacteriocin from *Lactobacillus plantarum* NC8. Appl Environ Microbiol.

[17] Nguyen HA, Nguyen TH, Nguyen TT, Peterbauer CK, Mathiesen G, Haltrich D (2012). Chitinase from *Bacillus licheniformis* DSM13: expression in *Lactobacillus plantarum* WCFS1 and biochemical characterisation. Protein Expr Purif.

[18] Nguyen TT, Nguyen HM, Geiger B, Mathiesen G, Eijsink VGH, Peterbauer CK, et al. Heterologous expression of a recombinant lactobacillal β-galactosidase in *Lactobacillus plantarum*: Effect of different parameters on the sakacin P-based expression system. Microb Cell Fact. BioMed Central Ltd.; 2015. p. 14. 10.1186/s12934-015-0214-8 PMC4358714 25880197

[19] Ng DTW, Sarkar CA (2013). Engineering signal peptides for enhanced protein secretion from *Lactococcus lactis*. Appl Environ Microbiol.

[20] Le Loir Y, Azevedo V, Oliveira SC, Freitas DA, Miyoshi A, Bermúdez-Humarán LG (2005). Protein secretion in *Lactococcus lactis*: an efficient way to increase the overall heterologous protein production. Microb Cell Fact.

[21] Cano-Garrido O, Seras-Franzoso J, Garcia-Fruitós E (2015). Lactic acid bacteria: reviewing the potential of a promising delivery live vector for biomedical purposes. Microb Cell Fact.

[22] Romero Pastrana F, Neef J, van Dijl JM, Buist G (2017). A *Lactococcus lactis* expression vector set with multiple affinity tags to facilitate isolation and direct labeling of heterologous secreted proteins. Appl Microbiol Biotechnol.

[23] Cano-Garrido O, Sánchez-Chardi A, Parés S, Giró I, Tatkiewicz WI, Ferrer-Miralles N (2016). Functional protein-based nanomaterial produced in microorganisms recognized as safe: a new platform for biotechnology. Acta Biomater.

[24] García-Fruitós E, Rodríguez-Carmona E, Díez-Gil C, Ferraz RM, Vázquez E, Corchero JL (2009). Surface cell growth engineering assisted by a novel bacterial nanomaterial. Adv Mater.

[25] García-Fruitós E, Seras-Franzoso J, Vazquez E, Villaverde A (2010). Tunable geometry of bacterial inclusion bodies as substrate materials for tissue engineering. Nanotechnology.

[26] Martínez-Miguel M, Kyvik AR, Kyvik AR, Ernst LM, Cano-Garrido O, Garcia-Fruitós E (2020). Stable anchoring of bacteria-based protein nanoparticles for surface enhanced cell guidance. J Mater Chem B.

[27] Sans C, García-Fruitós E, Ferraz RM, González-Montalbán N, Rinas U, López-Santín J (2012). Inclusion bodies of fuculose-1-phosphate aldolase as stable and reusable biocatalysts. Biotechnol Prog.

[28] Gifre-Renom L, Ugarte-Berzal E, Martens E, Boon L, Cano-Garrido O, Martínez-Núñez E (2020). Recombinant protein-based nanoparticles: elucidating their inflammatory effects in vivo and their potential as a new therapeutic format. Pharmaceutics.

[29] Torrealba D, Parra D, Seras-Franzoso J, Vallejos-Vidal E, Yero D, Gibert I (2016). Nanostructured recombinant cytokines: a highly stable alternative to short-lived prophylactics. Biomaterials.

[30] García-Fruitós E, Vázquez E, Díez-Gil C, Corchero JL, Seras-Franzoso J, Ratera I et al. Bacterial inclusion bodies: making gold from waste. Trends Biotechnol. 2012. p. 65–70. 10.1016/j.tibtech.2011.09.003 22037492

[31] García-Fruitós E, Villaverde A (2010). Friendly production of bacterial inclusion bodies. Korean J Chem Eng.

[32] Hrabárová E, Achbergerová L, Nahálka J. Insoluble protein applications: the use of bacterial inclusion bodies as biocatalysts. Methods Mol Biol. 2015. p. 411–22. 10.1007/978-1-4939-2205-5_24 25447879

[33] López-Cano A, Bach A, López-Serrano S, Aragon V, Blanch M, Pastor JJ (2022). Potential of oral nanoparticles containing cytokines as intestinal mucosal Immunostimulants in Pigs: a pilot study. Anim (Basel).

[34] Roca-Pinilla R, López-Cano A, Saubi C, Garcia-Fruitós E, Arís A (2020). A new generation of recombinant polypeptides combines multiple protein domains for effective antimicrobial activity. Microb Cell Fact.

[35] Pesarrodona M, Jauset T, Díaz-Riascos ZV, Sánchez-Chardi A, Beaulieu ME, Seras-Franzoso J (2019). Targeting Antitumoral proteins to breast Cancer by local administration of functional inclusion bodies. Adv Sci.

[36] Vázquez E, Corchero JL, Burgueño JF, Seras-Franzoso J, Kosoy A, Bosser R (2012). Functional inclusion bodies produced in bacteria as naturally occurring nanopills for advanced cell therapies. Adv Mater.

[37] Unzueta U, Seras-Franzoso J, Céspedes MV, Saccardo P, Cortés F, Rueda F (2017). Engineering tumor cell targeting in nanoscale amyloidal materials. Nanotechnology.

[38] Vandooren J, Van Den Steen PE, Opdenakker G (2013). Biochemistry and molecular biology of gelatinase B or matrix metalloproteinase-9 (MMP-9): the next decade. Crit Rev Biochem Mol Biol.

[39] Tran AM, Unban K, Kanpiengjai A, Khanongnuch C, Mathiesen G, Haltrich D (2021). Efficient secretion and recombinant production of a lactobacillal α-amylase in *lactiplantibacillus plantarum* WCFS1: analysis and comparison of the Secretion using different Signal peptides. Front Microbiol.

[40] Curiel JA, Rodríguez H, Acebrón I, Mancheño JM, De Las Rivas B, Muñoz R (2009). Production and Physicochemical Properties of recombinant *Lactobacillus plantarum* tannase. J Agric Food Chem.

[41] Geiger B, Nguyen HM, Wenig S, Nguyen HA, Lorenz C, Kittl R (2016). From by-product to valuable components: efficient enzymatic conversion of lactose in whey using β-galactosidase from *Streptococcus thermophilus*. Biochem Eng J.

[42] Kaswurm V, Nguyen TT, Maischberger T, Kulbe KD, Michlmayr H (2013). Evaluation of the food grade expression systems NICE and pSIP for the production of 2,5-diketo-D-gluconic acid reductase from *Corynebacterium glutamicum*. AMB Express.

[43] Sasikumar P, Gomathi S, Anbazhagan K, Selvam GS (2013). Secretion of biologically active heterologous oxalate decarboxylase (OxdC) in *Lactobacillus plantarum* WCFS1 using homologous signal peptides. Biomed Res Int.

[44] Liu Q, Jiang Y, Yang W, Liu Y, Shi C, Liu J (2020). Protective effects of a food-grade recombinant Lactobacillus plantarum with surface displayed AMA1 and EtMIC2 proteins of Eimeria tenella in broiler chickens. Microb Cell Fact.

[45] Fredriksen L, Mathiesen G, Sioud M, Eijsink VGH (2010). Cell wall anchoring of the 37-kilodalton oncofetal antigen by *lactobacillus plantarum* for mucosal cancer vaccine delivery. Appl Environ Microbiol.

[46] Kuczkowska K, Kleiveland CR, Minic R, Moen LF, Øverland L, Tjåland R (2017). Immunogenic properties of *lactobacillus plantarum* producing surface-displayed Mycobacterium tuberculosis antigens. Appl Environ Microbiol.

[47] Kuczkowska K, Mathiesen G, Eijsink VGH, Øynebråten I (2015). *Lactobacillus plantarum* displaying CCL3 chemokine in fusion with HIV-1 gag derived antigen causes increased recruitment of T cells. Microb Cell Fact.

[48] Fredriksen L, Kleiveland CR, Olsen Hult LT, Lea T, Nygaard CS, Eijsink VGH (2012). Surface display of N-terminally anchored invasin by *Lactobacillus plantarum* activates NF-κB in monocytes. Appl Environ Microbiol.

[49] Straume D, Axelsson L, Nes IF, Diep DB (2006). Improved expression and purification of the correctly folded response regulator PlnC from lactobacilli. J Microbiol Methods.

[50] Bhatwa A, Wang W, Hassan YI, Abraham N, Li XZ, Zhou T (2021). Challenges Associated with the formation of recombinant protein inclusion bodies in *Escherichia coli* and Strategies to address them for Industrial Applications. Front Bioeng Biotechnol.

[51] Rueda F, Gasser B, Sánchez-Chardi A, Roldán M, Villegas S, Puxbaum V (2016). Functional inclusion bodies produced in the yeast *Pichia pastoris*. Microb Cell Fact.

[52] Wang L (2009). Towards revealing the structure of bacterial inclusion bodies. Prion.

[53] Sriubolmas N, Panbangred W, Sriurairatana S, Meevootisom V. Localization and characterization of inclusion bodies in recombinant *Escherichia coli* cells overproducing penicillin G acylase. Appl Microbiol Biotechnol. 1997;373–8. 10.1007/s002530050943 9163951

[54] Rinas U, Garcia-Fruitós E, Corchero JL, Vázquez E, Seras-Franzoso J, Villaverde A (2017). Bacterial inclusion bodies: discovering their better half. Trends Biochem Sci.

[55] Gifre-Renom L, Carratalá JV, Parés S, Sánchez-García L, Ferrer-Miralles N, Villaverde A (2020). Potential of MMP-9 based nanoparticles at optimizing the cow dry period: pulling apart the effects of MMP-9 and nanoparticles. Sci Rep.

[56] Carratalá JV, Cano-Garrido O, Sánchez J, Membrado C, Pérez E, Conchillo-Solé O (2020). Aggregation-prone peptides modulate activity of bovine interferon gamma released from naturally occurring protein nanoparticles. N Biotechnol.

[57] Gifre-Renom L, Seras-Franzoso J, Rafael D, Andrade F, Cano-Garrido O, Martinez-Trucharte F (2020). The biological potential hidden in inclusion bodies. Pharmaceutics.

[58] Serna N, Sánchez JM, Unzueta U, Sánchez-Garcia L, Sánchez-Chardi A, Mangues R (2019). Recruiting potent membrane penetrability in tumor cell-targeted protein-only nanoparticles. Nanotechnology.

[59] Kopp J, Spadiut O. Inclusion Bodies: Status Quo and Perspectives. 2023; 2617:1–13. 10.1007/978-1-0716-2930-7_1 36656513

[60] Mierau I, Kleerebezem M (2005). 10 years of the nisin-controlled gene expression system (NICE) in *Lactococcus lactis*. Appl Microbiol Biotechnol.

[61] Axelsson L, Rud I, Naterstad K, Blom H, Renckens B, Boekhorst J (2012). Genome sequence of the naturally plasmid-free Lactobacillus plantarum strain NC8 (CCUG 61730). J Bacteriol.

[62] Aukrust T, Blom H (1992). Transformation of Lactobacillus strains used in meat and vegetable fermentations. Food Res Int.

[63] Cano-Garrido O, Garcia-Fruitós E, Villaverde A, Sánchez-Chardi A (2018). Improving Biomaterials Imaging for Nanotechnology: Rapid Methods for protein localization at ultrastructural level. Biotechnol J.

[64] Seras-Franzoso J, Peebo K, García-Fruitós E, Vázquez E, Rinas U, Villaverde A (2014). Improving protein delivery of fibroblast growth factor-2 from bacterial inclusion bodies used as cell culture substrates. Acta Biomater.

